# Atrial Fibrillation: The Year of 2021 in Review

**DOI:** 10.19102/icrm.2022.130108

**Published:** 2022-01-15

**Authors:** Mayank Sardana, Rahul N. Doshi

**Affiliations:** ^1^HonorHealth Cardiovascular Center of Excellence and Cardiac Arrhythmia Group, HonorHealth, Scottsdale, AZ, USA

**Keywords:** Ablation, atrial fibrillation, review

*“Progress is impossible without change, and those who cannot change their minds cannot change anything.”—George Bernard Shaw*

Electrophysiology has continued to progress on many fronts, certainly none more than how we treat atrial fibrillation (AF). As the most common arrhythmia worldwide,^1^ it deserves to get the most attention within our community. The last year has been no exception, with the release of some incredibly powerful data relating to all aspects of care, including ablation. The usual disclaimer applies—trials we discuss here are by no means an exhaustive list, as we chose to cover work with far-reaching implications.

## The ACTIVE-AF trial

Before we jump into the interventions for managing AF, how about a little workout? The Inflammatory Response as a Prognostic Factor of Recurrence of Atrial Fibrillation After Ablation (ACTIVE-AF) trial was presented at the European Society of Cardiology meeting in 2021.^2^ This was a randomized controlled trial investigating the impact of an exercise prescription in 120 patients with symptomatic paroxysmal or persistent AF. The participants in the exercise arm were enrolled in a supervised exercise program followed by an individualized weekly plan to follow at home with the goal of increasing aerobic exercise to 3.5 hours per week over six months. At 12 months of follow-up, the rates of AF recurrence were significantly lower in the exercise arm (60%) as compared to the control arm (80%). Furthermore, there was a significant difference in AF symptom severity in the exercise arm compared to the control arm. Overall, this study adds to lifestyle modification strategies, which greatly impact the burden and symptoms of AF (eg, weight loss, addressing sleep-disordered breathing, aerobic exercise, reducing alcohol consumption). While these generic recommendations for lifestyle modification are likely to benefit all patients with AF, addressing individual-specific triggers for AF can also impact the reduction of the AF burden as demonstrated in some of the trials we will discuss.

## The EAST-AF4NET trial and early treatment of atrial fibrillation

How we view AF has evolved rapidly over the last several decades, but we have long required a contemporary version of the classical Atrial Fibrillation Follow-up Investigation of Rhythm Management (AFFIRM) and Rate Control Efficacy in Permanent Atrial Fibrillation (RACE) trials.^3,4^ Despite the dozens of commentaries and subgroup analyses performed, the resounding message to the medical community was that rate control and oral anticoagulation were equivalent to rhythm control. Obviously, our treatment options for AF have evolved, but until the Early Treatment of Atrial Fibrillation for Stroke Prevention Trial (EAST-AF4NET),^5,6^ we still were without data supporting more aggressive treatment for AF rhythm control with implications for the general medical community. Data suggest that most patients with AF are not on rhythm control.^7^ However, most experts would agree that most hard endpoints in early randomized clinical trials of rate versus rhythm control were driven by hospitalizations, and rhythm-control strategies require more hospitalization given the need for monitoring for antiarrhythmic drug (AAD) initiation.

The now timeless observation of how “AF begets AF” has been supported by data ranging from benchtop investigations of the mechanisms of atrial remodeling to large-scale clinical trials. A Trial with Dronedarone to Prevent Hospitalization or Death in Patients with Atrial Fibrillation (ATHENA), which demonstrated the clinical benefit of oral dronedarone for AF, also reveals that early initiation of therapy results in lower progression from paroxysmal to permanent AF.^8^ Hence, the rationale for the EAST-AF4NET trial was to explore whether early rhythm-control treatment results in favorable outcomes compared to rate-control strategies. This was a randomized clinical trial comparing guideline-directed therapy with rate control and oral anticoagulation and rhythm control to treat symptoms of AF as per guidelines, versus early rhythm-control treatment consisting of AAD or ablation therapy. A total of 2,789 patients with AF for less than one year were randomized with a median 5.1 years of follow-up. The primary outcome was a composite of cardiovascular death, stroke, hospitalization from heart failure, or acute coronary syndrome and was reduced by 21% in the treatment group (*P* = .005). Stroke was reduced by 35%, and total mortality was reduced by 16%. This landmark clinical trial clearly demonstrates benefits in hard endpoints of a rhythm-control strategy early in the diagnosis of AF.

The benefits of early initiation of rhythm-control strategy were seen for both AAD therapy (dronedarone and amiodarone) and ablation therapy. There are recent trials suggesting that an ablative strategy is superior to AAD therapy. The Atrial Fibrillation Progression Trial (ATTEST) supports this notion. This was a randomized controlled trial of 322 patients with paroxysmal AF comparing ablative therapy with AAD therapy.^9^ The investigators demonstrated at three years of follow-up that only 2.4% of patients treated with ablation progressed to persistent AF, as compared to 17.5% of patients treated with AAD (*P* = .0009). Patients treated with ablation were 10 times less likely to progress to persistent AF. Benefits were seen at one year and persisted throughout the follow-up period.

Similarly, the STOP-AF First trial randomized 203 patients who had never had rhythm-control treatment initiated to either AF ablation with a cryoballoon strategy or AAD treatment with either class I or III drugs excluding amiodarone.^10^ They demonstrated a one-year success rate of 74.6% in the ablation group versus 45.0% in the drug group. The population treated had a mean duration of AF diagnosis of 1.3 years. The most used AAD was flecainide. Importantly, there were no differences in serious adverse events reported in the trial.

When taken together, these important clinical trials clearly support earlier rhythm-control treatment initiation and support the notion that catheter ablation when done safely is preferable as a first-line treatment. Early intervention in patients with AF may prevent or delay progression and results in better long-term management. This is supported by evidence that favorable structural remodeling associated with treatment is seen more with ablation when compared to AAD therapy. An important substudy from the Catheter Ablation vs. Anti-arrhythmic Drug Therapy in Atrial Fibrillation (CABANA) trial supports this notion. In the CABANA imaging substudy, approximately 200 patients had pre-procedural and follow-up imaging (mean, 100 days) with either cardiac computed tomography or magnetic resonance imaging (MRI) to quantify left atrial volumes and pulmonary vein (PV) diameter.^11^ The left atrial volume index decreased 52.9% in the ablation group versus 40.0% in the AAD group. This finding is consistent with prior evidence that catheter ablation is associated with favorable remodeling that may result in long-term clinical benefits such as arrhythmia burden and stroke prevention.

## CABANA substudies and specific groups treated for ablation

This last year provided a wealth of subgroup analysis from the landmark CABANA study, as the imaging substudy illustrates. The most powerful is the subanalysis of patients with heart failure.^12^ Seven hundred seventy-eight patients in the CABANA trial had a New York Heart Association (NYHA) functional class of II or more at the time of enrollment. Most of these patients had a preserved ejection fraction, with only 20% of patients having a left ventricular ejection fraction of less than 50%. The primary endpoint of the CABANA trial is a composite score of death, disabling stroke, serious bleed, or sudden death. In the intention-to-treat analysis, the ablation arm had a 36% reduction in the primary endpoint and a 43% decrease in mortality over the mean follow-up of 48.5 months. Thirty-seven percent of patients in the ablation arm demonstrated recurrence versus 58% with drug therapy at one year. Moreover, 35% of patients in the group had paroxysmal AF versus 65% who were persistent or long-standing persistent, suggesting that ablation despite a patient cohort with more advanced disease and a lower overall success rate (defined by one-year freedom from arrhythmia) still resulted in clinical benefit. AF burden in the ablation group was 7% versus 18% in the AAD arm. Much like the Catheter Ablation for Atrial Fibrillation with Heart Failure (CASTLE-AF) study in a reduced left ventricular ejection fraction population,^13^ the importance of reduction in AF burden seems to be a more meaningful clinical endpoint.

The CABANA trial also provides us with a large contemporary patient cohort to examine first-line ablation treatment in important groups traditionally undertreated or underrepresented in clinical trials. A subgroup analysis of the CABANA trial looking at differences in sex reveals important considerations in the treatment of women with AF.^14^ Despite women in the trial having more advanced symptoms (48% of women with Canadian Cardiovascular Society AF severity class 3 or 4 vs. 39% for men) and more heart failure (42% of women with NYHA class ≥ II vs. 32% of men), there were no differences between men and women in both treatment efficacy and adverse event rates. Men did have a higher percentage of freedom of AF at one year (66% in men vs. 56% in women). The available data support the notion that a treatment strategy utilizing ablation should not be withheld from women with AF.

A subgroup analysis of the minority population in the CABANA cohort revealed similar results.^15^ A total of 9.9% of patients in the CABANA trial were classified as ethnic or racial minorities and had a higher incidence of hypertension, heart failure, and decreased ejection fraction. However, this group had a 67% reduction in the primary composite endpoint and a 72% reduction in mortality that was statistically significant. The authors concluded that these improved outcomes were secondary to a poorer tolerance of AAD therapy compared to the non-minority patients. Forty-two percent of patients in the drug group were on amiodarone versus 17% in the ablation group.

Taken together, the subgroup analysis from the CABANA trial suggests that both women and ethnic or racial minorities do better with catheter ablation as compared to drug therapy—and historically have been undertreated.

## Pulsed-field ablation

Perhaps, the most exciting upcoming ablation strategy for AF ablation is pulsed-field ablation (PFA). Building upon the published research from 2020, this year, we saw several exciting developments for PFA. In one study, one-year outcomes were assessed for PFA in the patients with paroxysmal AF enrolled in the Safety and Feasibility Study of the IOWA Approach Endocardial Ablation System to Treat Atrial Fibrillation (IMPULSE), Safety and Feasibility Study of the FARAPULSE Endocardial Ablation System to Treat Paroxysmal Atrial Fibrillation (PERFCAT), and Expanded Safety and Feasibility Study of the FARAPULSE Endocardial Multi-ablation System to Treat Paroxysmal Atrial Fibrillation (PERFCAT II) trials.^16^ In this study, 110 trial patients who initially underwent PV isolation (PVI) using basket and flower petal PFA catheters underwent remapping at two to three months. Nearly 85% of PVs remained isolated on remapping (96% were durably isolated for the veins that received optimized biphasic PFA waveform). Nearly 79% of patients remained free of AF at one year of follow-up.

The mechanisms of PFA delivery (lasso configuration catheter, hybrid PFA/radiofrequency ablation lattice catheter, etc.) as well as our understanding of biophysics/lesion characteristics from PFA are rapidly evolving. There are several ongoing clinical trials that are investigating the safety and efficacy of PFA for AF ablation. The ADVENT study is a randomized study of PFA versus RF and cryoablation in patients with paroxysmal AF, the Pulsed Field Ablation to Irreversibly Electroporate Tissue and Treat Atrial Fibrillation (PULSED AF) study is an observational study in patients with paroxysmal and early persistent AF, and the INSPIRE study is an observational study in patients with paroxysmal AF (NCT04612244, NCT04198701, and NCT04524364, respectively).

## More ablation strategies: ice, alcohol, and the epicardium

As discussed already, the STOP-AF First trial demonstrated the superiority of ablation with the cryoballoon as the initial treatment for AF compared to therapy with AADs.^10^ This is corroborated by the similar Cryo-FIRST trial with a similar design.^17^ These two trials support the use of cryoballoon ablation as the first-line treatment for AF. Finally, the Cryoballoon Pulmonary Venous Isolation in Patients Referred for Typical Atrial Flutter Ablation (PAF-CRIOBLAF) trial illustrates the utility of empiric cryoballoon ablation in patients undergoing ablation of the cavotricuspid isthmus for typical atrial flutter.^18^ Patients were followed up for 24 months post-ablation, and the incidence of AF was 33% in the cryoballoon group versus 69% in the group receiving isthmus ablation alone. Taken together, these results suggest that in an early disease group, a straightforward PV strategy that can be done safely can halt the progression of early AF.

However, we continue to look for strategies that might increase our success rates for more advanced disease, as success rates are far from ideal. One novel approach is the use of ethanol injection to ablate the vein of Marshall (VoM). The Verifying the Effectiveness of the NUsurface^®^ System (VENUS) randomized clinical trial investigated the additive benefit of this approach to radiofrequency ablation alone.^19^ Three hundred fifty patients were randomized, with all patients getting wide area circumferential ablation and additional ablation at the discretion of the operator. One hundred eighty-five patients were randomized to receive VoM ablation, which was successful in 84% of patients. Single-procedure freedom from arrhythmia at 12 months was 49.2% in the combined group versus 38% in the standard therapy group (*P* = .04). Thus, targeting the VoM improved the outcomes of ablation in patients with persistent AF. The mechanism could be multifactorial, eliminating a potential focal source, targeting autonomic innervation, or facilitating achieving mitral isthmus block. A subsequent secondary analysis suggests that the latter is clearly related to improved outcomes.^20^

Another target of interest in addressing the AF burden in advanced disease is the left atrial appendage. The findings from the long-awaited aMAZE trial were recently presented at the American Heart Association Scientific Sessions.^21^ In this multicenter randomized trial of 610 patients with symptomatic persistent and long-standing persistent AF who were slated for their index catheter ablation, patients were assigned to PVI with or without concomitant percutaneous epicardial left atrial appendage occlusion using a Lariat^®^ device (SentreHEART, Redwood, CA, USA). Although the Lariat procedure had a high success rate for appendage occlusion (85% of patients with <1 mm residual communication at one year), there was no significant difference in freedom from atrial arrhythmias at one year. The trial findings are currently not published in a peer-reviewed journal, but some of the initial criticism arose from the protocol followed for catheter ablation—as adding posterior wall isolation or mitral isthmus linear ablation was not permitted. In addition, there is concern that the electrical isolation of the appendage using the Lariat^®^ device does not address the autonomic ganglia present along the ridge between the left superior PV and appendage. The currently enrolling Posterior Wall and Left Atrial Appendage Empiric Electrical Isolation for Non-paroxysmal Atrial Fibrillation (PLEA) trial should address some of these limitations as it seeks to determine the role of endocardial appendage isolation using RF in patients with non-paroxysmal AF (NCT04216667).

What if, instead of trying to fit a “one size fits all approach” for non-pulmonary venous targets, we targeted the regions of fibrosis specific to individual patients? The Efficacy of Delayed Enhancement Magnetic Resonance Imaging–guided Ablation Versus Conventional Catheter Ablation of Atrial Fibrillation (DECAAF-2) trial,^22^ which was presented at the European Society of Cardiology meeting in 2021, specifically attempted to answer this question. In this multicenter randomized trial, 843 patients with persistent AF were randomized to PVI plus imaging-guided fibrosis ablation (intervention group) or PVI alone (control group). The fibrosis was measured using late gadolinium enhancement on cardiac MRI, and the operators were instructed to either cover or encircle the areas of fibrosis in the intervention group. The intention-to-treat analyses did not reveal a significant difference in freedom from atrial arrhythmia at 12 months (57% in the intervention group vs. 54% in the control group). Interestingly, in analyses stratified by the degree of fibrosis, those with grade I or II fibrosis (<20% fibrosis) derived benefit from fibrosis-guided ablation compared to those with a higher degree of fibrosis. Also, as a cautionary note, higher rates of post-ablation stroke were noted in the patients undergoing imaging-guided ablation but were largely driven by those with a higher degree of fibrosis. Overall, this trial re-establishes PVI as the mainstream strategy in all patients with AF and, once again, supports intervention at earlier stages of fibrosis, ie, atrial remodeling.

## Ablation strategies: convergent approach

In patients with advanced disease, another approach that has been proposed is the combined surgical and catheter ablation approach. In the multicenter, randomized Convergence of Epicardial and Endocardial Ablation for the Treatment of Symptomatic Persistent AF (CONVERGE) trial, 150 patients with persistent and long-standing persistent AF were assigned in a 2:1 randomized manner to hybrid convergent ablation approach and catheter ablation.^23^ The hybrid group underwent epicardial ablation using a vacuum-assisted, unipolar radiofrequency ablation introduced via a subxiphoid/transdiaphragmatic approach followed by endocardial catheter ablation. The catheter ablation group underwent PVI with a roof line and cavotricuspid isthmus ablation line. Targeting complex fractionated atrial electrograms and additional ablation lesions was left to the operators’ discretion if the patients did not convert to sinus rhythm. The trial included patients with left atrial dimensions up to 6 cm (the average in the trial was 4.3–4.4 cm), and 42% of patients had long-standing persistent AF. Over a follow-up of 12 months, 67% of patients were free of atrial arrhythmias in the convergent group compared to 50% in the catheter ablation group. The immediate and intermediate safety profile of the surgical approach was good. The authors reported adverse effects in three of 102 patients within seven days after the procedure: one stroke, one excessive bleeding, and one excessive bleeding associated with late pericardial effusion. An additional five patients experienced adverse effects from days 7 to 28: three pericardial effusions, one phrenic nerve injury, and one transient ischemic attack. Interestingly, no major adverse effects were reported in the catheter ablation group.

Overall, this trial highlights a very important role of epicardial atrial surface in AF. In nearly 40% of patients, endocardial ablation does not lead to durable endocardial isolation of the posterior wall.^24^ The convergent approach leads to transmural ablation lesions while minimizing the risk of esophageal injury as the RF energy is directed toward the heart and away from the esophagus. Also, epicardial–endocardial dissociation and discordant wavefronts have been reported in persistent AF.^25^ Further refinement of epicardial mapping and ablation techniques (via the surgical or percutaneous approach) will help to improve our understanding of the AF mechanism and improve the ablation success rates.

## Conclusions

Overall, the year 2021 has been a critical one for the treatment of AF. Not only have there been significant advancements in technologies and techniques, but, more importantly, we have evidence supporting what we knew all along—AF is not a benign disease, and early and more effective interventions in the treatment of AF result in more favorable clinical outcomes.

We anxiously await what 2022 brings!
